# Critical Appraisal of Surface Electromyography (sEMG) as a Taught Subject and Clinical Tool in Medicine and Kinesiology

**DOI:** 10.3389/fneur.2020.560363

**Published:** 2020-10-26

**Authors:** Vladimir Medved, Sara Medved, Ida Kovač

**Affiliations:** ^1^Faculty of Kinesiology, University of Zagreb, Zagreb, Croatia; ^2^Clinic of Psychiatry and Psychological Medicine, University Hospital Center Zagreb, Zagreb, Croatia; ^3^Polyclinic Medical Body Balance, Zagreb, Croatia

**Keywords:** bioelectric signals, surface electromyography, teaching, clinical medicine, kinesiology, physiotherapy, rehabilitation

## Abstract

The characteristics and state of knowledge of bioelectric signals such as ECG, EEG, and EMG are initially discussed. This serves as the basis for exploration of the degree of scholastic coverage and understanding of the level of clinical acceptance of respective bioelectric signal subtypes during the last 60 or so years. The review further proceeds to discuss surface EMG (sEMG). The status of the field in terms of teaching and academic training related to sEMG is examined, and its clinical acceptance in several areas of medicine and kinesiology, including neurology, psychology, psychiatry, physiatry, physical medicine and rehabilitation, biomechanics and motor control, and gnathology, is evaluated. A realistic overview of the clinical utility of the measurement of sEMG signals and their interpretation and usage, as well as of perspectives on its development, are then provided. The main focus is on the state of the field in Croatia. EMG signals are viewed as “windows” into the function of the neuro-muscular system, a complex and hierarchically organized system that controls human body posture and gross body movement. New technical and technological means to enable the detection and measurement of these signals will contribute to increased clinical acceptance, provided current scientific, educational, and financial obstacles can be removed.

## Introduction

Since its beginnings around the mid-twentieth century, the field of surface electromyography (sEMG) (1) has evolved and became established as a measurement, analysis, diagnostics, and (motor) control tool. sEMG forms part of a standard palette of methods and technologies at the disposal of scientists and professionals in a number of disciplines such as neurology, psychology, psychiatry, physiatry, physical medicine and rehabilitation, kinesiology, biomechanics and motor control, and gnathology; each discipline exploits specific features of this technique. sEMG is a component of the broader EMG field that includes subcutaneous techniques. It also is a part of the biomechanics of movement and represents a unique vehicle for monitoring the function of the neuromuscular system. sEMG offers considerable robustness, non-invasiveness, and a global view of skeletal muscle function.

Here, our aim is to critically reflect on the position of this measurement technique in both educational curricula (of primarily medical doctors, physiotherapists, and kinesiologists) and the clinical environment. The starting point of this endeavor is a discussion of the relationship of sEMG with tools that rely on the acquisition and interpretation of other types of common bioelectrical signals. In particular, the status of the field in Croatia is considered.

## On The Nature of Bioelectric Signals and Their Interpretation

Changes in bioelectric potential originate in particular organs and organ systems. Because bodily tissues serve as an electrical conductor, potential changes generated spread through the body and reach the body surface, where they are amenable to detection by suitable technical means. In the following sections, the types of bioelectric signals most commonly recorded are succinctly depicted: an electrocardiogram (ECG) originates in cardiac muscle; an electroencephalogram (EEG) originates in the brain; and an electromyogram (EMG) originates in skeletal muscle(s).

The concept of membrane potential is central to bioelectric signal generation and conduction. The process of generation of change of equilibrium potential takes place at the cell membrane, which in a resting state maintains an electrical potential in the range of −40−90 mV (internal relative to external medium), the state of equilibrium being described by the Nernst equation (2, 3). When stimulated, under certain conditions, a change in potential across a membrane that reaches positive amplitude values, known as action potential, spreads along the membrane. Neural cells are electrically excitable and capable of conducting this electrical change (4). Further biophysical processes participate in transmission of electromagnetic fields, produced by bioelectric sources, through biological media (5).

### Electrocardiography (ECG)

The contracting heart muscle generates electrical potentials. Numerous studies have modeled the mechanical action of heart muscle and performed bioelectric imaging (6). Clark provided a condensed, but comprehensive, explanation of the bioelectric activity of the heart muscle based on its anatomy, the types of excitable cells that comprise its functional components, and the electrocardiogram, i.e., signal waveform of electrical potential changes detected at the outer surface of the body (3). The standard 12-lead electrocardiogram recording is generally used in clinical practice.

Because cardiac contractions are a repetitive and continuous process, the ECG exhibits a characteristic quasi-periodic waveform. Since the beginnings of the analysis of the bioelectric characteristics of the heart muscle, the ECG technique has become established as a reliable, important, and practical diagnostic tool. Clinical standards have since been developed for the diagnosis of arrhythmias, ischemias, and numerous other pathological conditions.

Beginning in the 1960s, computer-aided analysis of ECG was introduced (7), and, over time, large databases of signals were established, such as the MIT-BIH Arrhythmia Database^1^. The field of computerized ECG processing (8) has developed since; as a result, ECG has acquired prominence as a clinically indispensable tool in cardiology. The ECG produces patterns that can be empirically correlated, through visual inspection by an expert, with important aspects of health.

### Electroencephalography (EEG)

Since Hans Berger, a German psychiatrist, systematically analyzed the electrical activity of the brain for the first time, the EEG technique has gained prominence in neurology, contributing significantly to neurological diagnostics, and additionally proving useful in the fields of neuropsychiatry and psychology (3). The fluctuating potentials recorded represent a superposition of the field potentials produced by a variety of active neuronal current generators within the volume-conductor medium (3, 9). Extracellular potentials recorded from the cerebral cortex are to be interpreted.

Typical clinical EEG waveforms recorded using scalp electrodes (International Federation of EEG Societies 10-20 system) can be identified. Correlations exist with specific brain states (e.g., wakefulness, sleep) and specific pathologies associated with abnormal EEG waveforms. EEG frequency ranges are as follows: delta [below 3.5 Hz (usually 0.1–3.5 Hz)], theta (4–7.5 Hz), alpha (8–13 Hz), and beta (usually 14–22 Hz) (9).

Computer-aided EEG analysis has mainly been used for monitoring sleep and certain pathologic states, leaving the more difficult problem of diagnosis to the expert neurologist (7). Another potential field of application of EEG is, for example, as a brain-computer interface where EEG signals represent control signals for prostheses of extremities, as well as in a number of other areas such as the study of hypnosis.

### Electromyography (EMG)

The field of electromyography encompasses both surface and intramuscular EMG: here, however we focus exclusively on sEMG. Although sEMG provides a global view of skeletal muscle function, in principle, the analysis of multielectrode recordings enables assessment of the activity of individual motor units (MU) as well (10). In addition, multichannel sEMG offers the ability to study features of multiple muscle systems. The term multielectrode sEMG is used to identify EMG detection with multiple electrodes on the same muscle, while the term multichannel sEMG comprises several bipolar recordings in several muscles.

Muscular contraction is a mechanical event involving the transformation of metabolic energy into mechanical force and power. Leaving the mechanical aspects of muscular contraction aside (11), here we focus on the electrical features of the transmission of the signal (5). Muscle action potentials may be associated with chemical processes through which mechanical energy is released. To quote Katz (2): “In muscle, the action potential, traveling at a speed of a few meters per second, serves to produce sufficiently quick ‘mobilization' of the contractile apparatus in the interior of the cell.”

The total bioelectric signal of a muscle is the result of spatiotemporal summation of the activity of a large number of MUs, producing what is referred to as an interference pattern. Basmajian and De Luca presented a mathematical model of the myoelectric signal (1, 12, 13). Starting from the basic physiological processes that give rise to nerve and muscle action potentials, all higher anatomical levels of integration were considered, which finally yielded a total EMG signal. This anatomically complex situation can be considered, in fact, to be a sort of mapping of a spatial (3D) process into a 1D signal (14).

Individual fiber potentials—recording of which would pre-suppose needle detection—sum up to represent a motor unit action potential (MUAP). A wide range of neuromuscular disorders alter the MUAP waveform in different but characteristic combinations, the interpretation of which is the domain of neurological diagnostics (14, 15). A train of motor unit action potentials is referred to as a (MUAPT). Modeling of EMG signals is a rich and well-developed research area (16, 17).

**Figure 1:**
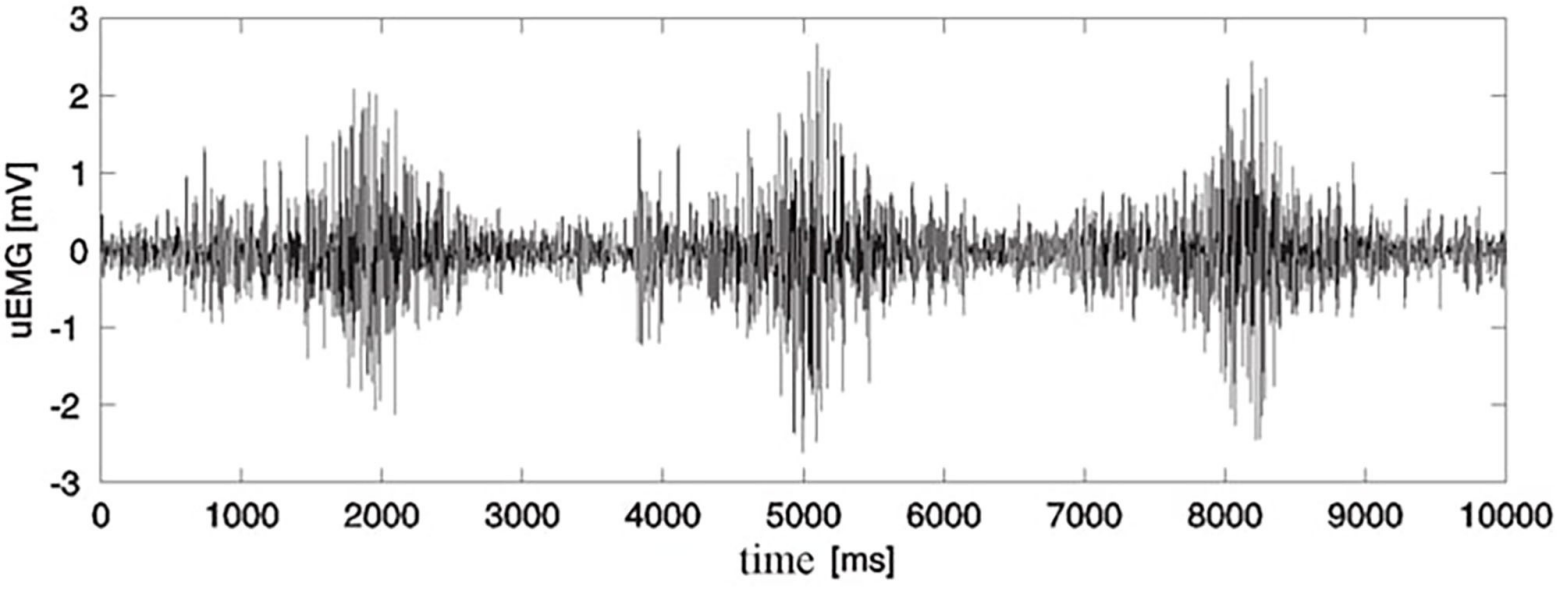
Raw surface EMG recording for three successive contractions of m. vastus medialis during extension-flexion exercise of the lower leg (18) (Permission was received from the Faculty of Electrical Engineering and Computing for the use of this image).

A typical sEMG signal is shown in Figure 1 [from (18)].

Standard bipolar detection technique is assumed. Fundamental concepts pertaining to EMG signal acquisition are comprehensively summarized in (19). sEMG signals are amenable to several methods of signal processing, both in time and in the frequency domain (20). A practical overview of the technical aspects of sEMG for clinicians is given in (21).

## Comment on Diagnostic Utility of Techniques Based on Bioelectric Signals and Their Clinical Acceptance

The diagnostic value of ECG and EEG has been established firmly in the respective medical fields of cardiology and neurology; in addition, these techniques may be conveniently used in less clinical realms such as health kinesiology (ECG) or in brain research (EEG). It is understood, of course, that these bioelectric indicators most often form part of more comprehensive measurement and evaluation schemes in addition to other clinical diagnostics data such as CT or NMR records and relevant laboratory diagnostic results. Therefore, ECG and EEG methods may certainly be considered to represent a reliable and standard component of the so-called evidence-based medicine approach.

Compared with ECG and EEG, the field of clinical use of sEMG might seem less developed and standardized. sEMG was first considered and accepted as a relevant quantitative indicator in physical and rehabilitation medicine. In this field of application, sEMG forms a standard component of an assortment of biomechanical measures relevant for monitoring, evaluation, and (motor) learning in the rehabilitation context (22). The goal was to make this field a part of evidence-based medicine, and effective in the management of physical disabilities. In kinesiology, sEMG is accepted as a standard component of the biomechanical evaluation inventory where gross body movements are concerned (23–27). In clinical gait analysis, for example, building on the pioneering work by the Berkeley group, sEMG has been used from the very beginning (23). It preceded the later inclusion of 3D kinematic measurement methods, which have progressed rapidly to offer high levels of precision and automatization in recent times, and are successfully combined with multichannel sEMG. The same holds true also for research applications of the analysis of human motion in kinesiology and sport science.

Essentially, and assuming a quantitative relationship between sEMG and muscle force [(20, 28–33)—consideration in a broader context], it can be asserted that sEMG has become a reliable non-invasive correlate of muscle force in addition to the more basic, but less “muscle selective,” means of assessing muscle force such as dynamometry, as well as to complementary methods such as mechanomyography. Recommendations for EMG measurements, processing (34–36) and interpretation (37) have been provided. As a result of advances in multielectrode sEMG [high-density sEMG (HD-sEMG)] recording techniques, further possibilities have emerged, both for research and for clinical applications (38–41).

## Scholastic Coverage of Bioelectric Signals

Measurement and interpretation of bioelectric signals, and of sEMG in particular, is a technical issue whereby a measurement method is applied to human subjects. Besides carrying diagnostic information, sEMG also may serve as a control variable (biofeedback, prostheses). It can therefore be explained and understood most thoroughly—both theoretically and practically—assuming a biomedical engineering (BME) perspective. We begin this section with a short comment on BME education, both worldwide and in Croatia, focusing later on education of medical doctors, physiotherapists and kinesiologists.

An inter- and multi-disciplinary approach to education is integral to the field of biomedical engineering. In Germany, between the two World Wars, and in the USA, especially after the World War II, independent university programs in the field of biomedical engineering were established. As a part of these efforts, DeClaris and Newcomb stated in the 1980s the need for biomedical engineering students to adapt traditional engineering knowledge of circuit theory toward systems theory in order to link them to physiological concepts more easily. Engineering analysis and design methods had to be adapted to the solution of problems related to biological systems (42). Biomedical engineering (bioengineering and clinical engineering, including medical physics) is today an established profession with a significant labor market. There are over 300 accredited biomedical engineering schools and university departments in the USA today, offering biomedical engineering programs at several levels up to a Ph.D.^2^. The field has undergone significant and rapid development worldwide.

At the beginning of the 1980s, at the Department of Biomedical Engineering, Johns Hopkins University School of Medicine, Baltimore, Maryland, USA, for example, the undergraduate curriculum for medical doctors already featured some basic concepts in the technical (engineering) sciences such as signal and systems theory and feedback control systems theory (43). These concepts were useful to medical students by enabling a better understanding of complex physiological systems, as has been recognized previously when explaining the necessity for teamwork in neurological diagnostics (44). We note that we are aware of the differences in university education in the USA and Europe for prospective medical doctors, wherein students in the USA, owing to the “pre-med” B.Sc. level study, have more opportunities to acquire broad-based knowledge.

In the current academic curricula, primarily for biomedical engineers, but also, to a lesser degree, for non-engineers (medical doctors, physiotherapists, kinesiologists), the issues of measurement and signal processing methods for all types of bioelectric signals (ECG, EEG, EMG, and others) are successfully covered. In addition to classical mathematical signal processing methods, which are commonly taught in engineering schools, numerous advanced methods, such as data mining, neural networks, artificial intelligence, and advanced statistics, are included. Further, there is no discrimination between applicability to ECG, EEG, and sEMG in this respect. It is understood that each of these signal subtypes is treated in accordance with the specificities of its domain of application. Numerous textbooks that cover these fields are available, e.g., Sornmo and Laguna (45), Begg and Palaniswarmi (46), Shiavi (47), Glaser (48), (an update of a classic Glaser and Ruchkin book by Academic: New York, from 1976), Akay (49); other books on sEMG and biomechanics specifically have already been pointed out in the previous section.

In Croatia, unlike in neighboring countries, no formal biomedical engineering educational programs are available to date; in contrast, in Italy and Slovenia, the BME field, including BME education, is highly developed. In Slovenia, the example of the Vodovnik group tradition from the late 1960s onward (50, 51) in the area of cybernetics in medicine may be considered, witnessed today by the organization of the forthcoming EMBEC 2020 symposium in Portorož, Slovenia^3^, with the Slovenian Society for Medical and Biological Engineering acting as a co-organizer. However, an initiative was launched at the University of Zagreb in 2012, namely the Coordination Committee for Development of Biomedical Engineering, which is formed of representatives from various departments and institutes. Only in 2019 was an adequate program designed with the goal to develop the first university program in BME, satisfying the standards for professional training and qualification (52). The pre-requisites for this kind of program have been designed through extensive activity at various university departments and institutes across Croatia, which have been loosely coordinated by the Croatian Biomedical Engineering and Medical Physics Society (CroBEMPS) based in Zagreb. The Society encompasses divisions of clinical engineering, medical physics, and biomechanics.

At present, teaching activity in the area of biomedical engineering and physics in Croatia is accomplished mainly through a number of graduate and post-graduate mandatory and elective courses, at several university departments. In the respective courses, in addition to teachers with an engineering background (and a science background where appropriate, e.g., in the case of physicists and chemists), teaching staff with a biomedical background participates, including those with clinical expertise.

The subject of sEMG is included in the curriculum of human locomotion study, with biomechanical approaches being pursued. At the University of Zagreb, the subject of locomotion is covered rather well, and includes courses at both the under- and the post-graduate level at several departments: the Faculty of Kinesiology, the School of Medicine, and the Faculty of Electrical Engineering and Computing, as well as at a couple of other institutions (53). Relevant programs are pre-dominantly internet accessible^4^^,^^5^^,^^6^^,^^7^^,^^8^.

Adequate laboratory facilities exist in Zagreb and in Pula, and, to a lesser degree, also in Split and Rijeka. Examples of undergraduate elective courses at the University of Zagreb are: Multisensor systems and locomotion^9^ and Measurement and analysis of human locomotion^10^. The teachers combine expertise from research and university teaching; furthermore, in the second mentioned course, expertise is additionally derived from clinical fields such as neurology, orthopedics, physical medicine and rehabilitation.

During undergraduate study at all medical faculties (Zagreb, Osijek, Rijeka, and Split) future physicians are introduced to EMG as an electrophysiological diagnostic method and its significance in treatment and rehabilitation in the fields of taught neurology, physical medicine, and general rehabilitation, as well as pediatrics^5^^,^^11^^,^^12^^,^^13^. Both surface and intramuscular EMG are covered. Academic specialization for future specialists of physical medicine and rehabilitation follows the rulebook on specialist training of doctors of medicine, with a specialization in physical medicine and rehabilitation, which is compatible with the program of the European Union of Medical Specialists (UEMS) and the European Board of Physical and Rehabilitation Medicine (54). During residency as well as post-graduate study in physical medicine and rehabilitation and in neurology, medical doctors become familiar with electromyoneurography (EMNG) as a diagnostic method, its method of implementation, interpretation of findings, and its implementation in a therapeutic program (drug therapy, physical therapy, or rehabilitation). In addition to lectures, clinical practice in the EMNG laboratory is a mandatory part of training. Medical doctors (neurologists, physiatrists, and pediatricians) who wish to practice EMNG attend training with experienced electromyography trainers at referral centers for the treatment of neuromuscular diseases, usually for a period of 3 months, after which they take an examination to become certified for independent work. University neurological clinics in Zagreb and Split offer an ongoing training possibility of this type. Further, in the curriculum of specialist post-graduate studies for physical medicine and rehabilitation, residents learn about the latest advances in and indications for the therapeutic use of EMG and biofeedback (54).

Physiotherapists in Croatia complete the physiotherapy studies at the University of Applied Health Sciences in Zagreb (Zdravstveno veleučilište; although named in English “University” this is a polytechnic level school in Zagreb, it is not a university level institution) after which they receive the title “Professional Bachelor of Physiotherapy (baccalaureus),” abbreviated as bacc. physioth. Additional education through the Specialist Professional Study for a period of 2 years confers the title of Bachelor of Physiotherapy or Master of Physiotherapy^14^. In course of their studies they acquire knowledge on sEMG and the skill of its application in biofeedback therapy as a part of rehabilitation. Also, they get elementary information on EMNG as a diagnostic method, as well as on application of sEMG as a subject in locomotion biomechanics course. They cannot automatically become assistants in EMNG laboratory, but may get additional education with a mentor, lasting 2 months, which they usually master easily due to good knowledge of functional anatomy of the neuro-muscular system.

At the Ph.D. level of studies, the subject of sEMG is taught primarily at the University of Zagreb (kinesiology, medicine, electrical engineering and computing), but is also included in a number of courses at other universities. Doctoral studies in kinesiology at the University of Zagreb may be taken as an example (55) where the subject of sEMG is covered in detail in a couple of courses on biomechanical aspects of human movement and exercise. Another particular example is the course Biomechanical and neurophysiological mechanisms offered by the Faculty of Electrical Engineering and Computing^15^. There is no Ph.D.-level study for physical therapists in Croatia to date; therefore, these professionals usually engage in Ph.D. programs in the fields of medicine or kinesiology. (Latest news tell about a so-called bridge program enabling them to pursue doctoral studies at the Faculty of Medicine, University of Rijeka and Faculty of Kinesiology, University of Zagreb, assuming several difference examinations.) In the absence of data on the actual numbers, it is not clear how many physiotherapists hold a doctorate in kinesiology or medicine, anyhow, they are amenable to pursue an academic career. It is worth mentioning that the University of Applied Health Sciences in Zagreb strives toward attaining a university level.

Employees of the Department for Rehabilitation and Orthopedic Devices University Clinical Hospital Zagreb participate in both undergraduate and post-graduate teaching of students of the School of Medicine, deliver classes for physiotherapists, occupational therapists, and nurses of the University of Applied Health Sciences, and collaborate in teaching delivered to students of the Faculty of Kinesiology, Faculty of Education and Rehabilitation Sciences, and Polytechnic (Tehničko veleučilište) in Zagreb. The Department further provides education for specialists in orthopedics, traumatology, and physical medicine and rehabilitation, as well as compulsory internships for B.Sc. degree students in physiotherapy, occupational therapy, and nursing. In offering education in the fields of prosthetics and orthotics, the Department collaborates with ISPO-Croatia (International Society for Prosthetics and Orthotics) and Human Study e.v., and with the Polytechnic in Zagreb.

A short overview of biomechanical research in Croatia, encompassing activities at the Faculty of Kinesiology and the Faculty of Electrical Engineering and Computing, University of Zagreb, and at the Peharec Polyclinic in Pula, appeared in the International Society of Biomechanics (ISB) Newsletter (56). The situation has since improved owing to the availability of partially equipped facilities for human motion measurement and analysis at the universities of Split and Rijeka.

There is, evidently, a striking difference between the training physiotherapists get compared to the other two categories; engineers and medical doctors. What would be needed in the first place is to have laboratory facilities better equipped (if equipped at all). Their curriculum includes courses on anatomy, physiology and biomechanics but in general there is a lack of well-equipped laboratory facilities. So, often, practical laboratory subjects such as sEMG measurement and analysis are organized through visits to a well-equipped laboratory of a collaborating institution (from which also visiting professors usually participate). So, although formally being a part of teaching program, various signal processing methods in time domain and in frequency domain can only be demonstrated and not practiced and thus acquired by students themselves. Laboratories, that are situated in other institutions (clinics, faculties) normally have a qualified professional staff including clinical engineers. Basic biomechanics and sEMG in physiotherapy schools is typically thought by a couple of teachers: a physiotherapist and an electrical or computer engineer.

Our experiences, and of some of our colleagues indicate that the acceptance of physiotherapy students of modern technology is good and that with better teaching conditions they would accept these, sometimes rather requiring technical protocols, and profit from it. There is no doubt that this kind of improvement in working conditions would have positive long-term consequences on the work of future physiotherapists, and that their clinical competences would also improve. In general, at present, adequate courses and upgrades for professors of physical therapy, on the subject of sEMG and other relevant medical technology, would be useful.

## Overview of the Clinical Acceptance of sEMG

There is no doubt that sEMG is a valuable research vehicle. In this section we address typical applications of sEMG suitable for clinical use. Although each of these applications possesses a capacity for a clinical method, the same is being realized in various degrees, depending on methodological, financial and/or other issues. There are rather stringent requirements for a clinical method: it should possess reliability, validity, sensitivity and specificity, all features being subject to verification by appropriate clinical testing and confirmation by relevant statistics. The clinical acceptance of sEMG is a key issue addressed in this paper. We explore the contribution of sEMG data to diagnostic, evaluation, treatment, (motor) control, and/or (motor) learning procedures taking place, primarily, in hospital wards where sEMG measurement equipment is available, because these factors determine its clinical acceptance. In addition, the equipment must have a satisfactory level of user-friendliness to be accepted by the staff.

In general, the neuromusculoskeletal system is amenable to a number of measurement techniques, including “muscle-centered” ones such as dynamometry, mechanomyography, ultrasound, and thermography. In the broader context, standard neurophysiological, physiological (physiology of activity, i.e., exercise physiology and sports medicine), and biomechanical methods are available. Electromyography is one of the well-established “muscle-centered” techniques. Although needle EMG (NEMG) has undisputed value in the neurological diagnostics of muscular diseases, it will not be discussed here, and we continue with the overview of sEMG applications.

In 2000, The American Academy of Neurology stated that more than 2,500 original articles, reviews, and books investigating the utility, techniques used, and clinical application of sEMG had been published (57). As a diagnostic measure, sEMG has been reported to be inferior to NEMG for evaluation of patients with neuromuscular disorders because of its limited spatial resolution, susceptibility to mechanical artifacts, and tendency for cross-talk between adjacent muscles. The authors considered sEMG an acceptable tool for kinesiologic analysis of movement disorders, and also found it useful in differentiating many types of tremors, myoclonus, and dystonia, for evaluating gait and posture, and performing psychophysical measurements of reaction and movement time (57). Because several technological advancements have been made since, we discuss typical methods and procedures in operation today where sEMG is used as a suitable quantitative tool for evaluation, diagnosis, and/or treatment of a particular human health condition or assessment of motor performance level. The clinical prominence of methods is of interest. We also refer, in particular, to the situation in Croatia.

### Polysomnography (PSG)

A laboratory-based nocturnal polysomnography (PSG) method involves simultaneous recording of multiple physiologic variables related to sleep and wakefulness. PSG is the most commonly used test in the diagnosis of abnormalities of sleep and/or wakefulness, and can directly monitor and quantify the number of respiratory events (obstructive, central, or complex) and the resultant hypoxemia. In addition, it is useful in treating sleep disorders from a psychiatric or neurologic (sleep-related epilepsy) viewpoint. Assessment of sleep stages requires EEG, electrooculography (EOG), ECG, pulse oximetry, respiratory effort measurement (thoracic and abdominal), end tidal or transcutaneous CO_2_, sound recordings to measure snoring, continuous video monitoring, and sEMG. One sEMG channel, usually chin or mentalis and/or submentalis, is used to record atonia during REM sleep or lack of atonia in patients with REM-related parasomnia. To assess bruxism, sEMG electrodes can be placed over the masseter. sEMG analysis of intercostal and abdominal muscles may be conducted to determine effort during respiratory events. sEMG recording for monitoring of limb muscles (tibialis anterior) is used for assessing periodic limb movements and restless legs syndrome (58, 59).

In Croatia, the PSG method is routinely used in both adults and children, in neurology clinics and pediatric clinics of clinical hospital centers in Zagreb, Rijeka, Split, and Osijek. Furthermore, it is used in the clinic in which pulmonary diseases are treated of the University Hospital Center Zagreb (Jordanovac) (60), a pediatric clinic in Zagreb, a pediatric hospital for pulmonary diseases in Zagreb (Srebrnjak), psychiatric hospitals (Rab, Vrapče), and a number of private polyclinics in Zagreb, Split, and Rijeka. As far as we are informed, there are no problems (technical, methodological) with implementing sEMG within polysomnography and using the available information in clinical context.

### sEMG in Biofeedback

Biofeedback is a self-regulatory procedure through which patients are given feedback that enables them to develop control over their physiological functions through the provision of real-time data (61). Scientists and clinicians have used sEMG feedback as a tool when treating various medical disorders. Early studies on neurological disorders, such as torticollis, and in neurologically damaged patients have been reported (62–64). Psychosomatic diseases and functional disorders have also been targeted as a potential area for therapeutic interventions based on sEMG.

The first therapeutic applications of sEMG in biofeedback (sEMG-B) in psychiatry were carried out in the late 1960s. The goal of the treatment was to achieve relaxation, as a principal or adjunct mean of therapy (65). One study in 1991 evaluated the relationship between task performance and extrapyramidal effects of medication among psychiatric patients in a short-term stay psychiatric hospital, using sEMG-B. (66). Patients performed worse than healthy subjects in the psychophysical judgment task, showing impaired ability to make accurate psychophysical judgment, i.e., difficulty learning a biofeedback task that requires this skill.

sEMG signal is the most common physiological variable monitored using biofeedback, and is used in a variety of disorders such as tension headache, chronic pain, spasmodic torticollis, and temporomandibular joint dysfunction. EEG feedback, which is also called neurofeedback, is used in attention deficit hyperactivity disorder (ADHD) and epilepsy, and is increasingly the focus of research and other applications. Other commonly monitored variables are used when the aim of biofeedback is to reduce sympathetic arousal (heart rate, respiration rate, skin surface temperature, skin conductance, and heart rate variability).

Efficacy ratings for biofeedback training for various medical conditions have been reported in a previous study (67). Based on the Task Force of the Association for Applied Psychophysiology and Biofeedback and the Society for Neuronal Regulations' criteria, five levels of evidence-based clinical efficacy are defined: not empirically supported, possibly efficacious, probably efficacious, efficacious, efficacious and specific. sEMG has shown to be both efficacious and specific for female urinary incontinence; further, sEMG has been shown to be efficacious for anxiety, ADHD, chronic pain, constipation in adults, epilepsy, headache in adults, hypertension, motor sickness, Raynaud's syndrome, and temporomandibular disorders (TMDs). In alcoholism or substance abuse, arthritis, diabetes mellitus, fecal incontinence, headaches in children, insomnia, traumatic brain disorder, urinary incontinence in males, and vulvar vestibulitis, sEMG has been shown to be probably efficacious (67). Furthermore, the efficacy of biofeedback in psychiatric disorders specifically was confirmed for treatment of chronic anxiety, generalized anxiety disorder, panic disorders and post-traumatic stress disorder (PTSD) (68).

sEMG-B finds important applications in the field of rehabilitation: the signals are fed back to the patient, allowing patients to self-identify their muscle activity (69, 70). This is the most widely used and well-understood method of biofeedback, and has been shown to be useful in both musculoskeletal and neurological rehabilitation. The majority of biofeedback therapy is applied in the treatment of upper limb and lower limb motor deficits in neurological disorders. It can be used to either increase activity in weak or paretic muscle, or to facilitate a reduction in tone in a spastic one. It has been used since the early 1970s to improve gait, treat swallowing disorders, and enhance upper extremity function (71, 72). In daily clinical practice, sEMG is most frequently used in the treatment of weak or paretic muscles due to peripheral nerves injuries, as part of physical therapy, in order to increase their activity and strength. sEMG has additionally been used in the post-operative rehabilitation of surgically treated nerve injuries, as well in non-operated ones. Before visual or even palpable contractions occur, sEMG-B can provide valuable feedback to the patient and guide rehabilitation focused on sensorimotor re-education. However, complete therapeutic effectiveness can be achieved when the patient's voluntary muscular contractions occur (even in trace). By using sEMG-B therapy, both the therapist and the patient are provided with precise information about desirable and undesirable strategies for motor task execution aimed at improving muscle force and fine motor skills (73).

sEMG-B has demonstrated its usefulness in improving muscular torque and muscle recovery, as an addition to a conventional exercise program (74) such as that targeting the quadriceps femoris muscle after knee surgery or anterior cruciate ligament reconstruction (75), meniscectomy (76), arthroscopic partial meniscectomy (77), and in the treatment of pain due to excessive muscular tension (73). In order to reduce muscle tonus, sEMG-B has been used in spastic patients, both for the rehabilitation of hemiplegic adults after stroke, and in children with cerebral palsy (CP). Several studies have evaluated the effectiveness of biofeedback treatment on gait function in children with CP, e.g., sEMG-B of triceps surae muscle activity during gait, which may be used for improving gait symmetry in these patients (78, 79). Another group of authors has demonstrated the potential benefits of sEMG-B in conjunction with exercise in maximizing hand function in hemiplegic patients (80–82) or suggested that treadmill gait retraining augmented with sEMG-B facilitates improvements in gait function in post-cerebrovascular accident patients (83). These studies therefore indicate that sEMG-B is effective for post-stroke rehabilitation.

There is indeed a large number of medical conditions for which sEMG-B can be applied. In addition to those already mentioned, spinal cord injuries and low back and neck pain can also be addressed using this tool (84). The essence of the technique is illustrated well by the following statement: “A biofeedback device can be thought of as a sixth sense which allows the subject to ‘see' or ‘hear' physiological functions. Biofeedback can also be described as a ‘psycho-physiological mirror' providing subjects with a way to monitor the physiological signals produced by the body and learn from them to self-regulate a targeted pattern of physiologic functioning” (85).

Gallina et al. (84) introduced multi electrode recording techniques into the field of sEMG-B, making this technique potentially more intuitive and specifically adapted to the patient. Research in the field is oriented toward clinical application (86).

sEMG-B is applied in clinical praxis in Croatia, in all adequately equipped physical therapy units across the country. As far as we are informed, this is maybe the most traditional use of sEMG in medicine and is successfully being applied across the country to aid in various disturbances and diseases.

### sEMG in the Evaluation of Muscle Coordination

Although still pending clinical application, the evaluation of muscle coordination by means of sEMG signals deserves to be mentioned. This kind of application is pre-dominantly research-based in nature, and involves the measurement, processing, and correlation of myoelectric signals of several muscles that are co-active in performing a certain movement. A possible use is in sports research, where smoothed (full-wave rectified and low-pass filtered) sEMG signals that represent correlates of muscle forces may be used to quantify the degree of muscular coordination when performing a motor task, as has been performed in artistic gymnastics for example (27, 87). This approach has been used for quantification of the skill and performance level of a specific movement pattern, and has demonstrated the possibility for use of multichannel sEMG signals as indicators of the co-ordination patterns of multiple muscle forces associated with particular movements. Further, it offers possibilities for monitoring the progress in motorics during the course of particular diagnostics and/or treatment procedures in rehabilitation medicine.

Another possible application of muscle coordination evaluation is in the control of prostheses (section Myoelectric Prostheses).

Taborri et al. (88) performed a systematic review of the feasibility of muscle synergy outcomes in clinics, robotics, and sports. The muscle synergy concept underlies the ability of the central nervous system to control a large variety of muscles, via their simultaneous activation rather than individually, thus reducing the dimensionality of muscle control. It represents the continuation and further development of motor control concepts, which were originally conceived by Bernstein (the Moscow School of Biometrics) who developed a hierarchical multilevel model of organization of the system controlling voluntary movement and proposed the topic of many degrees of freedom in motor control (89). A number of studies have demonstrated that muscle synergies are robust across different tested conditions, within a period of a day as well as between days; within a single subject, and between subjects that have similar demographic characteristics. Taborri et al. (88) provide information for diagnosis or pathology assessment in clinics. A review of the available papers published between 2006 and 2017 was performed, taking into consideration only publications that provided results that were potentially useful for improving neuromuscular diagnosis and rehabilitation assessment for locomotion, balance, and upper limb functions. The pathologies addressed were locomotion and balance disturbances, CP, spinal cord injury, Parkinson's disease, stroke (quantifying abnormalities in modular muscle coordination; quantifying effects of therapy on muscle synergies; elucidating neural mechanisms of post-stroke muscle coordination), upper limb function, and pain. This indicates great, yet insufficiently explored possibilities that multiple EMG signals serve as “windows” into the function of the neuro-muscular system.

To the best of our knowledge, in Croatia, the above-mentioned issues are not currently being investigated, nor have they achieved clinical application at present. Our own experience includes the series of research projects investigating biomechanical and neuro-muscular aspects of complex movements, including sEMG correlates of (loco)motor skill. We have explored the issue on sportive movement patterns as a model of entrainment and skill acquisition (27, 87). Although having arrived at suitable quantitative measures of skill for a particular movement pattern, we did not standardize it to be usable as a clinical measure. Potential fields of application are neurorehabilitation and control of neuroprostheses.

### sEMG-Based Evaluation of Local Muscle Fatigue

**Figure 2:**
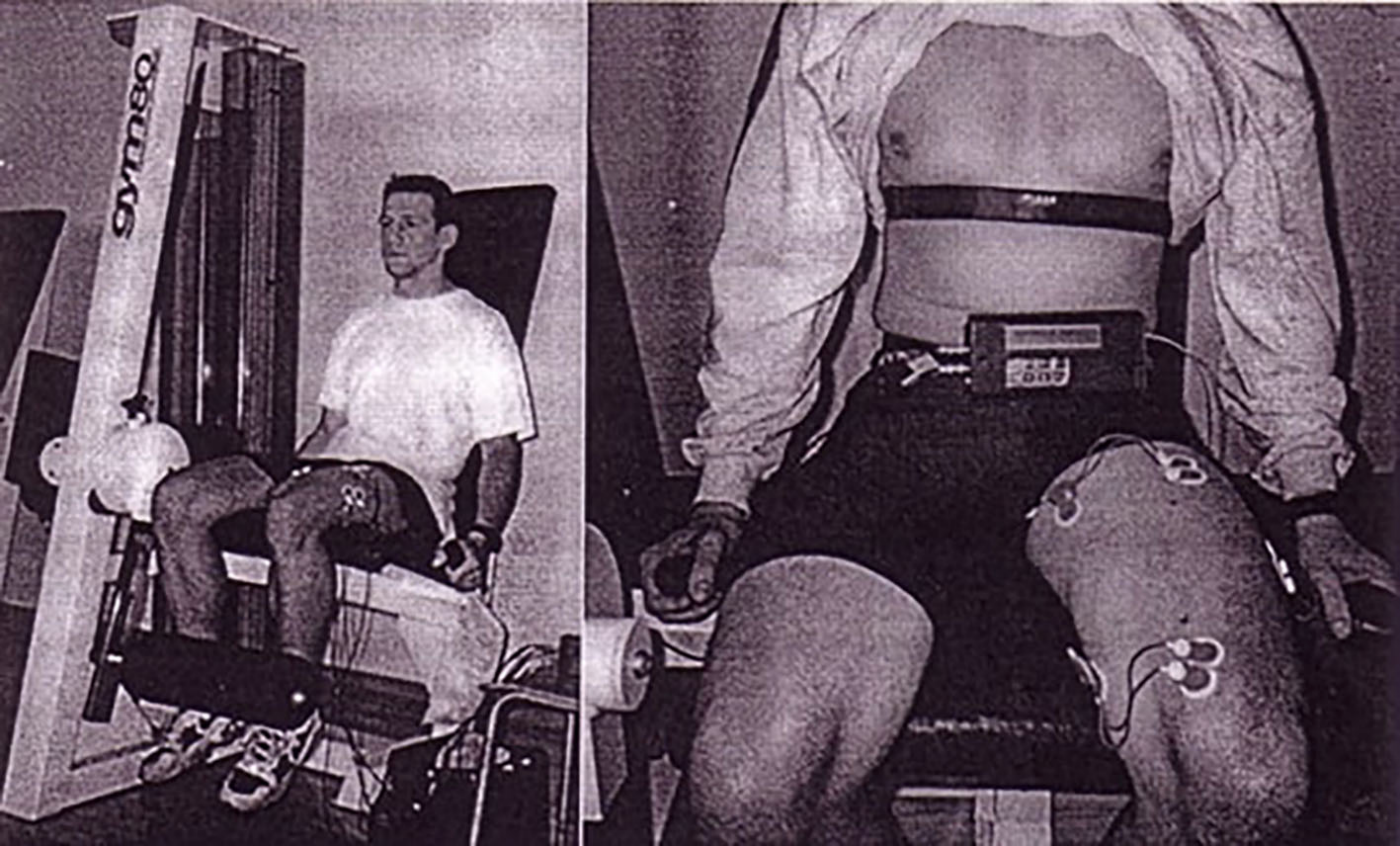
Investigation of local muscle fatigue during lower extremity extension-flexion exercise under loading (18). Subject ready to perform repetitive extension-flexion exercise of lower extremity under loading to induce fatigue (Permission was received from the Faculty of Electrical Engineering and Computing for the use of reproduced image. Written informed consent was obtained from the experimental subject for the publication of identifiable image).

**Figure 3:**
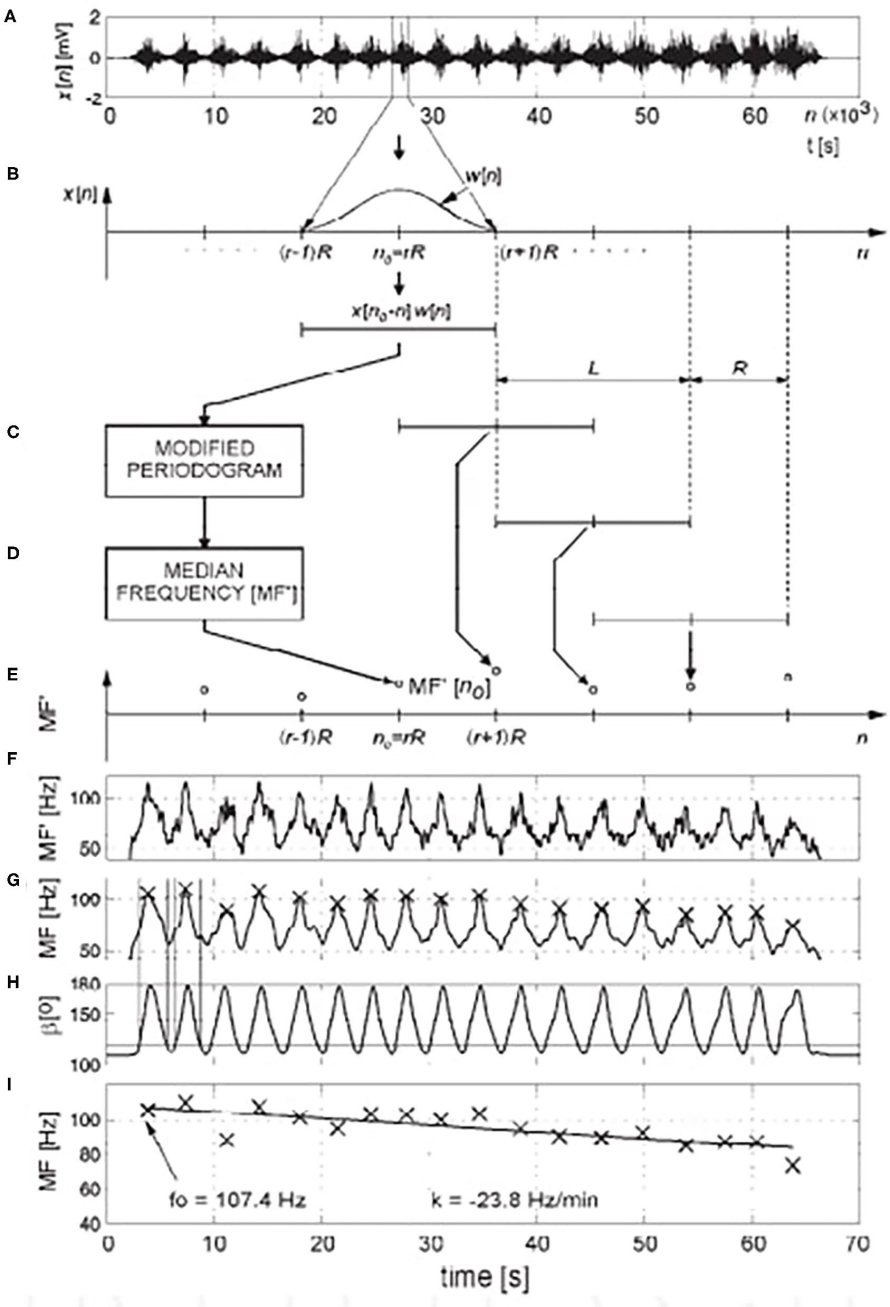
Investigation of local muscle fatigue during lower extremity extension-flexion exercise under loading (18). Myoelectric signal spectral analysis for quantification of muscle fatigue during dynamic contractions: **(A)** sEMG signal x[*n*], raw data; **(B)** extracted data, using window sequence w[*n*] of length L, with shift of R samples **(C–E)** estimation of median frequency (MF') using modified periodogram of windowed sequence, **(F)** course of median frequency (MF'), **(G)** after low-pass filtering, maximum values of MF during each contraction were calculated, **(H)** limits of contractions were calculated using shaft angle data, **(I)** the slope of the regression line (*k*, expressed in Hz/min) that fit maximum values of MF in a least-square sense was used as a fatigue index. From the regression line, the frequency at the beginning of exercise (f_0_) was calculated (Permission was received from the Faculty of Electrical Engineering and Computing for the use of reproduced image).

A distinct area of application of sEMG signals is in evaluating local muscle fatigue. This feature is based on the inherent property of a myoelectric signal to reflect the physiological status of fatigue in a muscle during muscular work. The phenomenon was first noticed as early as 1912 (90), and has subsequently been investigated and quantified extensively (18, 91, 92). Appropriate signal processing procedures, pre-dominantly in the spectral domain, have been defined, and shown to be able to quantify fatigue during static as well as dynamic contractions, as well as during electrically stimulated muscle activity (1, 13, 33, 71, 92, 93). We also additionally investigated the phenomenon during isometric and dynamic contractions primarily in the course of physical exercise and sports performance, and developed signal processing algorithms (27, 94–98) (Figures 2, 3).

Further development of the signal processing methodology (99) is aimed toward clinical applications, in particular, in the pathology of low back pain (100–102). Another example of our research in the field of muscle fatigue is in the sport of table tennis, to be applied in the optimization of sports training (96).

In Croatia, despite being rather practical for application, the sEMG technique, to our knowledge, is not yet routinely applied in clinical settings for evaluation of muscle fatigue, whether in a rehabilitation or a sports training context. We believe this is partly attributable to difficulties related to the standardization of mathematical signal processing methods to evaluate fatigue under conditions of dynamic contractions. Our own experiences include using the method in various movement patterns related to physical exercise and sports activities, as referred to before. Croatian BME group did have plans to design a practical and versatile method, supported by smart phone and suited for outdoor applications. So one may conclude that this method fails shortly to satisfy requirements of a clinical muscle fatigue evaluation method. But, we are of the opinion that perspectives are rather good.

### sEMG in Clinical Gait Analysis

The general fields of kinesiology, biomechanics, and motor control have witnessed the widespread use of sEMG as an important indicator in the quantitative characterization of movement patterns, both healthy and pathological. In combination with kinematic and kinetic measurement data, multichannel sEMG forms a standard component of instrumental setups in motion analysis laboratories aimed at measuring human posture and movement, including gait. It can typically be found in hospitals, orthopedic wards, pediatric clinics, physical medicine and rehabilitation settings, sports medicine clinics, as well as in research institutes and university departments (23, 103–106).

**Figure 4:**
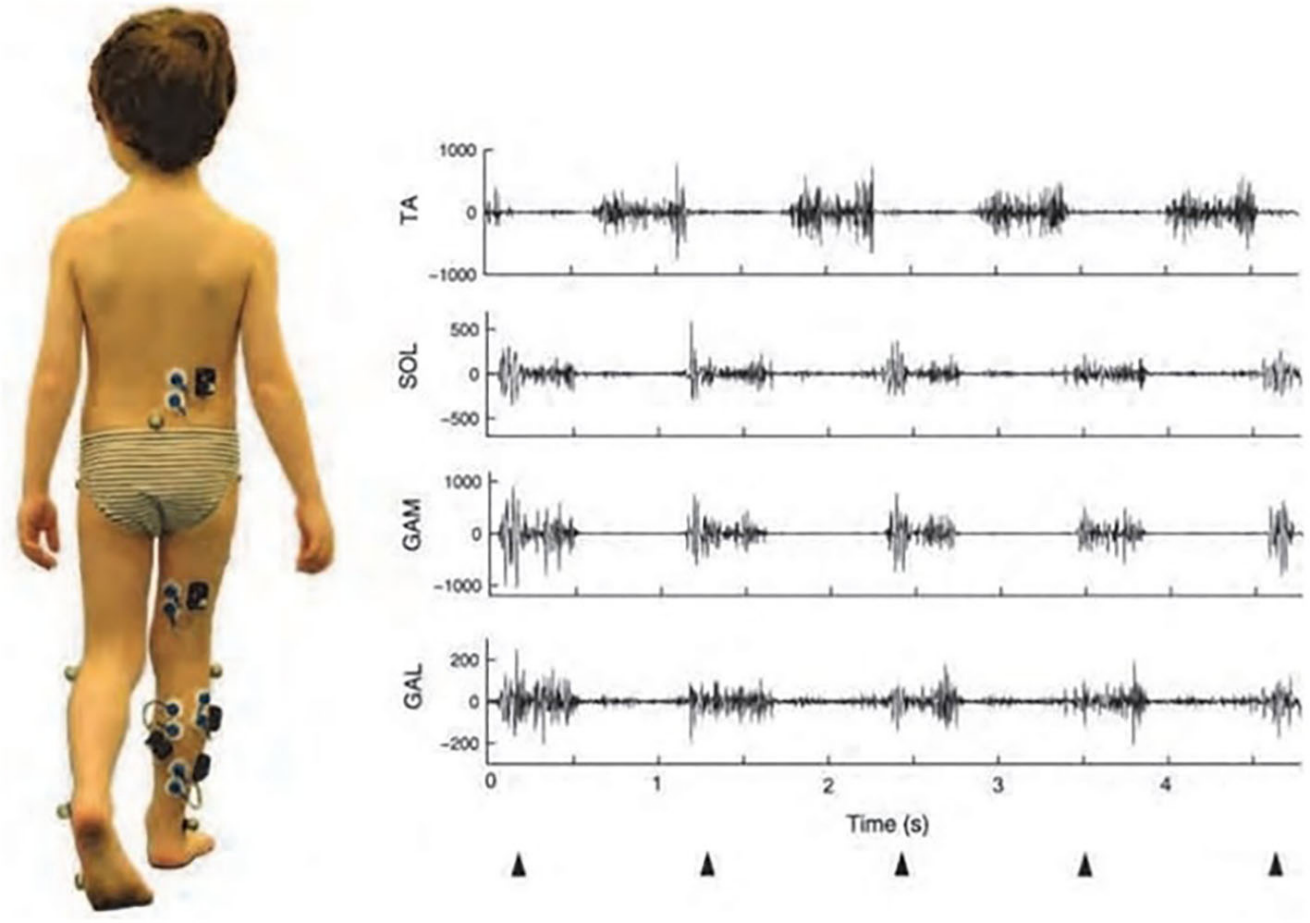
sEMG recording in a 5-year-old child during gait, as a component of a comprehensive gait report. The picture on the left shows the electrodes and the self-powered cases, each of which was provided with a pre-amplifier and antenna for independent transmission of myoelectric signals. Traces on the right-side are illustrative examples of EMG activities recorded from the tibialis anterior (TA), soleus (SOL), gastrocnemius medialis (GAM), and gastrocnemius lateralis (GAL) during a tiptoe walking task (107). Triangles below the diagrams indicate first contacts of the foot with the ground (Permission obtained from Clinical Biomechanics for the use of the image).

In the referred literature, books, as well as in hundreds of papers, the clinical value of gait analysis is undisputedly documented, although uncertainties and limitations do exist that are well-known to the biomechanics and motor control community; however, a review of these is beyond the scope of our paper. sEMG information, which is typically obtained in the form of an 8 or 16 channel telemetrically obtained record, certainly bears its value and represents a component of a valid comprehensive gait report (107) (Figure 4). In the applications like this one, sEMG enables elucidating muscle involvement and co-ordination in performing a task of walking. In pathological situations manifested with gait abnormalities multichannel sEMG adds valuable information.

Whether a particular gait analysis requires sEMG data is specific to the problem at hand; sometimes, only kinematic and kinetic information suffices.

Critical appraisal of the situation in Croatia, however, shows that clinical gait analysis is only provided by the Peharec Polyclinic in Pula. Although the necessary equipment and expertise exist at the Faculty of Kinesiology, University of Zagreb, and despite adequate experience in research applications: kinematics and kinetics in (108), kinematics, kinetics, and sEMG in (109), for example, gait analysis has not been implemented at the clinical level as of yet. One of the reasons are financial and organizational constraints, i.e., the lack of qualified staff required to operate such a facility. To meet international standards, the operation of this kind of unit requires appropriate staff, available at a full-time and/or a part-time basis, with multidisciplinary competencies. For a clinical gait analysis laboratory, such staff would ideally include a director, manager, gait analyst, biomechanist, technician, clinician, and clerical officer (105). But, due to the existence of a number of clinical centers, and with about one million of people gravitating to the Zagreb area, there is a high probability that soon clinical gait analysis might be realized and included into the pallete of diagnostic methods in health care and in sport science.

### Myoelectric Prostheses

The modern field of design, development, and application of prostheses of extremities is highly technologically complex. Here, we do not provide a comprehensive overview, but explore some important points. In the context of myoelectric prostheses, sEMG undoubtedly plays an important role: since cybernetics was first conceptualized by Norbert Wiener, sEMG forms a natural connection between a biological and technical sub-system of a “man-prosthesis” system. Childress (110) illustrates the historical aspects of development of powered limb prostheses up to the 1980s, while examples of modern approaches are presented in (111–115).

Motorized prostheses are typically controlled with sEMG data recorded on the residual muscles of amputated limbs. However, the residual muscles are usually limited, especially after above-elbow amputations; as a result, sufficient sEMG signals for the control of prostheses with multiple degrees of freedom cannot be obtained. Signal fusion is a possible approach that may be applied to resolve the problem of insufficient control commands, wherein some non-EMG signals are combined with sEMG signals to provide sufficient information for motion intensity decoding. One possible solution is to combine sEMG and EEG, in order to improve the control performance of the upper limb. Prosthetic hands differ in their complexity and components with some offering different grip patterns (adjustable by external means). Although myoelectric components of the prosthetic hand, wrist, and elbow are available, and various combinations of myoelectric-controlled components with body-powered components to control shoulder and/or elbow function can be created, optimal results are achieved for below-elbow amputation. For proper control of the prosthetic hand, the evaluation for possible muscle or nerve damage of the stump must be performed; then, the selection and calibration of the most effective electrode site on the clear silicone test socket fitting must be carried out using an adequate tester or sEMG analysis.

With regard to the status of the field in Croatia, other parts of the former State have, in the past, made greater creative contributions to the field; these include The Belgrade Hand by Tomović (110) and the previously mentioned Ljubljana Vodovnik group as examples. A series of meetings titled “Advances in External Control of Human Extremities” were held in Opatija and Dubrovnik; in Opatija, Norbert Wiener himself once participated.

In Croatia, the field subsequently progressed through implementation of available technical solutions, and myoelectric prostheses have been used since the 1990s, during the rehabilitation of war amputees at the Department for Rehabilitation and Orthopedic Devices University Clinical Hospital Zagreb in Zagreb, using Otto Bock products (Otto Bock HealthCare GmbH^16^). Subsequently, these prostheses became available in other rehabilitation units as well (Osijek, Rijeka) (116).

We may critically value current status of the field of myoelectric control in Croatia as a branch providing standard routine service in realms of health care, with—as far as we know—no efforts in pursuing novel solutions potentially possible based on modern technology. But, as in recent years collaboration between university institutes and small electronic and mechatronic firms rose, it is possible that some advancements to the field will come.

### sEMG in Gnathology

Gnathology is the study of the masticatory system, including physiology, functional disturbances, and treatment. In this field, Klasser and Okeson (117) have provided a comprehensive review of the literature regarding the scientific support for the use of sEMG in diagnosing and treating TMDs. Articles on the clinical utility of sEMG based on reliability, validity, sensitivity, and specificity of the results were included. The gold standard used to identify the presence or absence of TMD, or one of its subcategories, involves a comprehensive evaluation of the patient's history and clinical examination supplemented, when deemed appropriate, with imaging. After critically reviewing the relevant biological variables, the authors concluded that measurement of sEMG is inherently problematic, with many limitations, and thus has questionable value. The clinical use of sEMG in the diagnosis and treatment of TMDs has been found to be of limited value when one considers reliability, validity, sensitivity, and specificity of the measurement standards. sEMG does not appear to contribute any additional information beyond what can be obtained from the patient history, clinical examination, and, if needed, appropriate imaging. In conclusion, while sEMG has been found appropriate as a research tool, its clinical usefulness has been found restricted mainly to the area of biofeedback training.

In Merlo et al. (118), a sEMG-based method was devised wherein muscle contraction onset periods were computed by a wavelet-based method for muscle on-off detection, which proved suitable for clinical applications and is completely automated. It was applied in (119) in a clinical study of chewing problems in children and their correction. sEMG was recorded simultaneously with chewing kinematics, and, after processing, the data were used for evaluating coordination between the bilateral masseter muscles. Authors reported the correction of the malocclusion with a functional appliance, resulting in a favorable change in the neuromuscular control of chewing among patients, who recovered a normal-like coordination between the masseter muscles during chewing and a significant reduction of the reverse chewing patterns.

Although the issue of the clinical usefulness of sEMG in the aforementioned applications remains controversial, bioengineering and biomechanical approaches seem promising in offering viable solutions.

As already mentioned in section sEMG in Biofeedback, sEMG-B has been successfully used in TMDs (67).

Although we are aware of a long tradition of using sEMG within the School of Dental Medicine, University of Zagreb, to the best of our knowledge, it has not been applied in clinical praxis in Croatia to date; numerous private dental offices across Croatia do not use sEMG either. Based on personal observation of the first author, knowing both electronics and dental medicine experts in Zagreb University based institutions, a part of lack of success in developing valid clinical methods is in a rather conservative attitude that “engineers have to design the equipment and implement signal processing techniques, while medical doctors (doctors of dental medicine in this case) alone have to use these equipment.” The field of BME attains a more inter-disciplinary attitude however.

### Future Prospects

**Figure 5:**
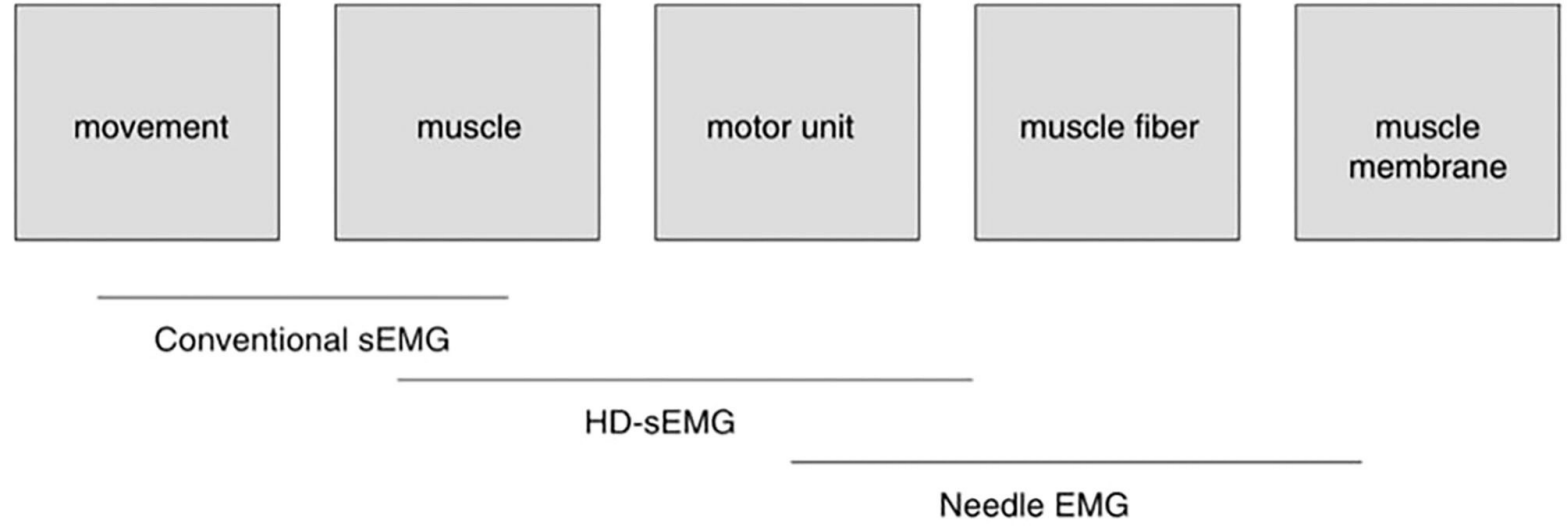
The scope of the various EMG techniques. Conventional bipolar sEMG, with one bipolar electrode pair over each muscle, is mainly used in movement studies. It yields information for muscle activity in different muscles simultaneously. The development of HD-sEMG, a technique that utilizes multiple electrodes on each muscle, has additionally made it possible to extract information at the single motor unit (MU) level. With HD-sEMG, information on muscle–fiber conduction velocity (MFCV) can be used to supplement the information at the muscle–fiber level obtained by needle EMG [from (39)] (Permission obtained from J EMG Kinesiol for the use of the image).

Important aspects of the development of sEMG technology are multielectrode (HD-sEMG) recording techniques. Although these techniques have been available for some time already (38–41), they are still rather avant-garde, and have not, to our knowledge, achieved widespread application as yet, however, they offer remarkable new possibilities. One direction is to explore, by mathematical analysis of measured signals, the functioning of particular MU in a muscle, thus providing a potential complement to the needle electrode detection technique and advancing neurological diagnostics. Although this sounds like an ambitious goal, it is supported by references [(10), as mentioned in section Electromyography (EMG), and (39)]. Drost et al. have already provided a systematic review of the clinical applications of HD-sEMG in 2006 (39). Clinical studies of muscle fatigue, motor neuron disease, neuropathies, myopathies (mainly in patients with channelopathies), spontaneous muscle activity, and MU firing rates have been reported. In principle, HD-sEMG allows the detection of pathological changes at the MU level, especially changes in neurogenic disorders and channelopathies. The authors described the status of the field at that time as being in the pre-clinical stage. Figure 5, which is taken from (39), elegantly illustrates the domains of application of different types of EMG detection. It is evident that HD-sEMG is applicable at the MU level.

Aside from the novel electrode technology mentioned here, one has to emphasize the technological development in the field of the acquisition systems. Small, wireless amplifiers have been developed that can be integrated with the detection system and interfaced with smart devices. This, although requiring certain versatility in operation and use (which, we believe, is easily surmounted with praxis) enables flexible applications of measurement equipment outdoors and in different milieus.

An interesting further ramification of the methods of signal detection is presented by Inzelber and Hanein (120) who report on novel technologies using printed electronics-type electrodes. Being ultrathin, these electrodes are designed so that the contact area be maximized, the contact impedance lowered, and movement artifacts simultaneously reduced. High-density printed sEMG has emerged as a non-invasive method for acquiring precise information related to muscle activation by increasing the electrode number and enabling data analysis schemes. The authors describe various practical applications, such as in sleep research, electrooculography, and REM recording; among these, the most interesting may be the measurement of sEMG from the cheek and eyebrow regions to detect emotions. This latter application should enable the detection of facial expressions as, ultimately, a potential marker of neuropsychiatric conditions. Therefore, printed electrodes on soft substrates, together with advanced analysis schemes of the acquired data, provide a facile and inexpensive tool for, potentially, objective mapping of neurophysiological abnormalities. Additional sensors, such as those measuring temperature and skin conductivity, may further enhance the performance of printed films. In addition to improving both printed technology and data analysis methodology, contributions to improved diagnostics, evaluation of treatment efficacy, and enhanced research possibilities of neuropsychiatric disorders may be facilitated. Ideally, such systems would enable automatic feedback and screening of normal vs. pathological conditions.

Another promising application is shown in (115) where HD-sEMG is used in Duchenne muscular dystrophy, a degenerative disorder, to enable thorough spatiotemporal analysis and pattern recognition of HD-sEMG signals providing the efficient control of exoskeleton, an active orthosis, in efforts to assist individuals in hand/wrist motor control. The ultimate aim is to enable functional solutions for performing daily living activities. The authors refer to bipolar (low-density) sEMG as a clinical golden standard control of robotic devices.

Our concluding remark on the future of sEMG focuses on man-machine interfacing possibilities in rehabilitation technologies (111). There are representative examples of the use of sEMG signals for device control purposes in neurotechnology, in three main areas of neurorehabilitation: replacement, restoration, and neuromodulation. In these examples, either data-driven or model-driven approaches can be used for processing the sEMG and generating control signals to external devices, prostheses, using advanced prosthesis control schemes. The possibilities of combining sEMG with subject-specific musculoskeletal models, allowing the establishment of an improved interface with the subject compared with that offered by traditional sEMG processing and movement analysis techniques, are promising. This is an approach compatible with the methodology introduced by Scott Delp, in the 1990s, that is well-known to the biomechanics community. It resides on a theoretical and experimental basis made available at the time for creating faithful mathematical models of the muscle-tendon complex (121), leading to computer-supported quantitative and graphics-based solutions to simulate the action of the neuromusculoskeletal system of a particular individual (122). This approach, which adds to classical inverse dynamics, enables detailed and realistic biomechanical modeling and simulation possibilities of complex neuromuscular systems and has been broadly implemented until our days, in research and clinical applications (123).

## Conclusion

The two main goals of our paper were as follows: first, to evaluate the scholastic coverage and second, to explore the degree of clinical acceptance of sEMG relative to ECG and EEG. We have summarized current knowledge in the field and provided an overview of the state of the art of technology and instrumentation. Our overview has been international in its approach, as well as specifically focused on the status of the field in Croatia.

The scholastic coverage, through teaching and training, of the areas discussed is only partially adequate, we believe, both worldwide as well as in Croatia. Academic training for all categories of students, i.e., physiotherapists, kinesiologists, medical doctors, engineers, consists of teaching basic knowledge on technical methods of recording, and processing the signals in question, with the goal of using these empirical data, along with other available variables and/or data, in intended research and clinical applications where it can be interpreted and used to solve a problem. Of course, when teaching engineering students, a more fundamental grasp of hardware/software knowledge is to be pursued as they as future professionals will be acting not only as users but also as designers and constructors of new equipment. The biomedical engineering approach is assumed, reflecting the inter- and multi-disciplinary nature of the field. This approach is prominent in existing curricula, as reported in section Scholastic Coverage of Bioelectric Signals, depending on the availability of appropriate laboratory facilities where signal recording equipment can be used and shown to students. The transfer of knowledge is possible, provided adequately trained staff are available. There is, of course, always room for improvement and inclusion of novel teaching tools. In addition to classical teaching methods, in a classroom and in a laboratory, online materials and courses, incorporating popular video clips for showing procedures and exercises, may be used.

Clinical acceptance of both ECG and EEG methods is superior and undisputed, as briefly stated in sections On the Nature of Bioelectric Signals and Their Interpretation and Comment on Diagnostic Utility of Techniques Based on Bioelectric Signals and Their Clinical Acceptance. The degrees of clinical acceptance of the sEMG method vary according to specific applications. There are many potential applications of this technique, as has been concisely discussed in section Overview of the Clinical Acceptance of sEMG. For these methodologies to be further implemented in Croatia and other countries, certain scientific as well as economic/organizational pre-requisites must be fulfilled. The scientific pre-requisites are 2-fold, comprising a research and a teaching component. The research component comprises the potential to conduct applied, clinical studies to validate and standardize applications of sEMG in the clinical environment. To achieve this, both equipment and manpower must be available, in the form of multidisciplinary research groups capable of securing research grants. The importance of proper staffing of clinical units and biomedical institutes is to be underlined at this point. This comprises having competent leadership in these institutions which requires interdisciplinary education to pursue clinical studies with the goal of developing and introducing new clinical methods. The importance of this “human” factor cannot be overemphasized.

Further, the applied component of research consists of the development of innovations (university-industry collaboration)—it is assumed that there should be a demand for such products—and presumes transfer of developed products to the market. At the University of Zagreb, Faculty of Electrical Engineering and Computing, an innovation center, namely the Inovacijski centar Nikola Tesla (ICENT)^17^ is being conceptualized, with a number of laboratories where engineering Ph.D.-level staff are to be employed. The ICENT project encompasses several institutes, including the Institute for Biomedical Engineering, which includes five laboratories; among these, the most relevant to the sEMG field being the Laboratory for Biomechanics and Laboratory for Biomedical Instrumentation.

Economic and organizational issues are a matter of broader social and even political endeavors supported by the relevant State ministries of health, science and the economy.

In regard to the appropriate teaching and education of primarily clinical staff, we believe that the acceptance of sEMG by the staff (physical therapists and kinesiologists, typically) is generally good. We speculate that EMG information may be more easily and intuitively interpreted by non-engineers than data obtained using technically more complex apparatus like CT or NMR. New trends in the presentation of information using intuitive and visually attractive displays will increase user-friendliness and further contribute to knowledge acquisition. However, a basic limitation is a fact that sEMG instrumentation, despite not being very expensive in comparison with many other technical methods in medicine, is still not available in all working environments where it could be useful. Through our teaching experience, we have witnessed a genuine interest in the features and potential of sEMG when introduced and explained for the first time.

The limitation of our study is that the assessment of the status of the field in Croatia has not been based on objective documented data on the adoption of sEMG instrumentation across institutions. We cannot conclude whether, at the level of the State, evidence of biomedical instrumentation exists in institutions such as hospital wards, university laboratories, and research institutes. Although some initiatives have been made in this direction by the State ministries of science, education, and health, these have not been implemented, to our knowledge. The effective implementation of such endeavors would be indispensable in this context.

Furthermore, we did not organize and conduct a survey among users of the equipment to assess their perceptions of the instrumentation and methodology; such data would be very informative.

We have based our statements on our subjective knowledge and our academic professional contacts, and the concepts explored were defined by our fields of expertise, namely electronic and biomedical engineering and biomechanics research and teaching; research and clinical work in psychiatry; and research, teaching, and clinical work in physical medicine and rehabilitation.

## Author Contributions

VM, SM, and IK conceived the manuscript. VM coordinated the activities of preparing the manuscript and mainly contributed to the biomedical engineering and kinesiology aspects. SM contributed to medical and clinical aspects, primarily in psychology, psychiatry, and neurology. IK contributed to medical and clinical aspects, primarily in physical and rehabilitation medicine, neurology, and myoelectric control. All authors contributed to the scholastic aspects, and wrote and approved the final version of the manuscript.

## Conflict of Interest

The authors declare that the research was conducted in the absence of any commercial or financial relationships that could be construed as a potential conflict of interest.

## Footnotes

^1^https://physionet.org/content/mitdb/1.0.0/

^2^https://www.educationnews.org/career-index/biomedical-engineering-schools/

^3^www.embec2020.org

^4^https://www.kif.unizg.hr/en/study

^5^https://mef.unizg.hr/studiji/diplomski/integrirani-preddiplomski-i-diplomski-studij-medicine/nastavni-plan-i-program

^6^https://mse.mef.unizg.hr/medical-studies-in-english/curriculum

^7^https://mef.unizg.hr/studiji/poslijediplomski/doktorski/phd-programme-in-english

^8^https://www.fer.unizg.hr/en/study_programs

^9^https://www.fer.unizg.hr/en/course/msal

^10^https://mse.mef.unizg.hr/medical-studies-in-english/curriculum/courses?studij=Medicina%20(na%20engleskom%20jeziku)&kol=3416&kolegij=Measurement%20and%20Analysis%20of%20Human%20Locomotion&pid=156

^11^https://www.medri.uniri.hr/hr/nastava.html

^12^http://www.mefos.unios.hr/index.php/hr/studij/sveucilisni-integrirani-preddiplomski-i-diplomski-studij-medicine

^13^http://www.mefst.unist.hr/studiji/integrirani-studiji/50

^14^as documented in: https://www.zvu.hr/?lang=en

^15^https://www.fer.unizg.hr/en/course/banm_a

^16^https://www.ottobockus.com/prosthetics/upper-limb-prosthetics/

^17^https://www.icent.hr/en/
